# Cadmium in Selected Organs of Game Animals from Areas with Different Degrees of Industrialisation and Its Intake by Human Consumers

**DOI:** 10.3390/ani14020305

**Published:** 2024-01-18

**Authors:** Małgorzata Bąkowska, Bogumiła Pilarczyk, Agnieszka Tomza-Marciniak, Renata Pilarczyk, Jan Udała

**Affiliations:** 1 Department of Animal Reproduction Biotechnology and Environmental Hygiene, West Pomeranian University of Technology in Szczecin, Klemensa Janickiego 29, 71-270 Szczecin, Poland; bogumila.pilarczyk@zut.edu.pl (B.P.); agnieszka.tomza-marciniak@zut.edu.pl (A.T.-M.); jan.udala@zut.edu.pl (J.U.); 2 Laboratory of Biostatistics, West Pomeranian University of Technology in Szczecin, Klemensa Janickiego 29, 71-270 Szczecin, Poland; renata.pilarczyk@zut.edu.pl

**Keywords:** cadmium, liver, kidney, roe deer, red deer, wild boar

## Abstract

**Simple Summary:**

A primary cause of heavy metal pollution in the environment is human economic activity associated with increased industrialisation and urbanisation, agricultural development, transport, and energy production. The present study determines the degree of contamination by cadmium (Cd), a heavy metal, based on its concentrations in the organs of animals living in regions of Poland. Our findings indicate that Cd is present in the highest concentrations in the southern regions of Poland, where the mining, energy, metallurgical, and steel industries are located. Surprisingly, high Cd concentrations were also found in the northeastern region, which is generally considered to be unpolluted.

**Abstract:**

The aim of this study was to determine the concentration of cadmium (Cd) in the organs of game animals from areas with different degrees of industrialisation and to assess its intake following the consumption of the livers of these animals. The material for this study consisted of liver and kidney samples taken from roe deer (*Capreolus capreolus*), red deer (*Cervus elaphus*), and wild boar (*Sus scrofa*) from five regions differing in land use and industrialisation. Compared to the kidneys, the liver was an indicator of the current state of the environment and Cd intake, while the kidney was used to assess the long-term accumulation of Cd in the body. The cadmium concentration in the samples was determined by inductively coupled plasma atomic emission spectrometry. The cadmium concentrations of the organs were found to vary according to geographical location, with the highest levels being observed in Southern Poland, i.e., areas characterised by the highest degree of industrialisation and the presence of heavy industry. However, high Cd concentrations were also reported in the northeastern region. Examples of excessive cadmium levels in the kidneys were noted in all tested regions. Cd intake by humans was evaluated by calculating the estimated daily cadmium intake, and this was compared with the tolerable weekly intake (TWI). The TWI was only exceeded by children frequently consuming wild boar liver from the southwest region.

## 1. Introduction

Cadmium (Cd) is a heavy metal that is toxic at any concentration and not biodegradable in the environment. It can accumulate to high levels in the body; however, its physiological role is unknown [1]. It is released into the environment as a result of both natural processes and human economic activities. European Environment Agency data indicate that Poland is the leading country in heavy metal emissions in Europe. Between 2005 and 2021, EU countries saw a 42% decrease in Pb emissions, a 47% decrease in Hg emissions, and a 37% decrease in Cd emissions compared to 1990. However, despite a general reduction in heavy metal emissions, Poland had the largest share of Pb, Cd, and Hg emissions in Europe in 2021, alongside Germany and Italy [2].

Cadmium accumulation in the soil is extremely dangerous. The soil is a fundamental element involved in the circulation of chemical elements and is the main link in the soil–plant–animal–human trophic chain. The Cd concentrations of soils generally depend on its concentrations in the bedrock; however, the level of Cd in soil is strongly influenced by human activities (mainly transport and energy production) and can be present in significant quantities. The level can also be increased by agricultural development and the use of phosphate fertilisers and plant protection products [3,4,5]. In Poland, soil contamination is often observed in industrialised areas, in metal ore mining and processing areas, near metallurgical and smelting plants, and land used for transport and agriculture [6,7]. A Polish study by Wdowczyk et al. [8] found the mean Cd concentration in soil to increase from 0.7 to 3.4 mg/kg between 1995 and 2020. It is also important to note that while Cd is immobilised in soils with neutral and alkaline pH, it changes to a mobile form as the pH decreases [9]. An important link in the movement of elements from the soil to animal and human tissues is constituted by plants. Plants take up Cd through the root system and leaf blades, with the amount depending on the concentration in the environment [10].

Most metals tend to bioaccumulate [11,12]. Likewise, environmental contamination with Cd results in its bioaccumulation in tissues, and in parenchymal organs, particularly the liver and kidneys. As Cd readily accumulates in the cortical part of the kidney and liver, the concentrations in these tissues are used as markers for assessing the degree of environmental contamination [13]. Numerous scientific papers have demonstrated that free-living animals, while remaining fully integrated into the environment, can act as bioindicators of pollution by heavy metals and other elements [14,15,16,17,18,19,20,21].

Significant variations in Cd level can be observed in the organs of game animals from areas with different levels of industrialisation; this indicates a relationship between the level of environmental pollution and the presence of this element in the animals.

The aim of this study was to determine the concentration of Cd in the organs of game animals from areas with different degrees of industrialisation, and to assess its intake following the consumption of the livers of these animals.

## 2. Materials and Methods

### 2.1. Location and Characteristics of the Study Area

**Figure 1:**
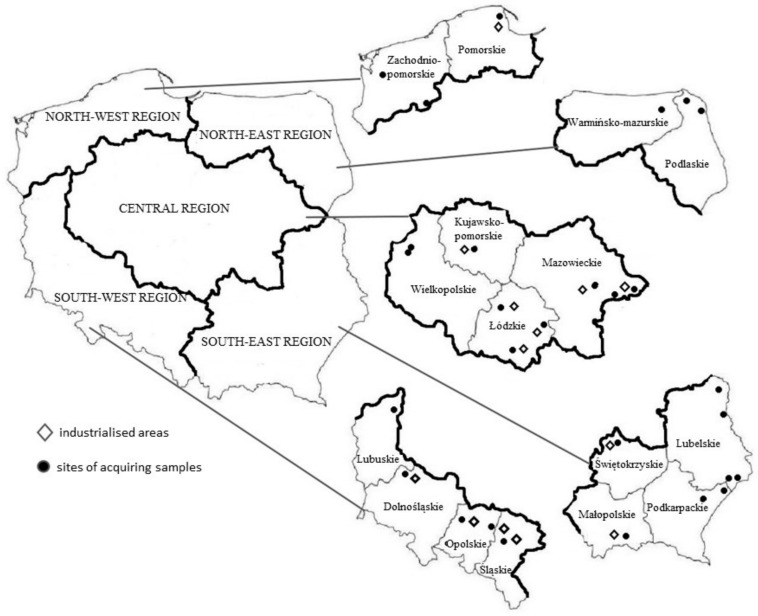
Locations of sample collection in accordance to geographic regions and industrialised areas.

Samples were obtained from the 16 voivodeships of Poland; these were divided into 5 regions for the purposes of the study: a northwest region (Zachodniopomorskie and Pomorskie Voivodeships), a northeast region (Warmińsko-Mazurskie and Podlaskie Voivodeships), a central region (Wielkopolskie, Kujawsko-Pomorskie, Mazowieckie, and Łódzkie Voivodeships), a southwest region (Lubuskie, Dolnośląskie, Opolskie, and Śląskie Voivodeships), and a southeast region (Lubelskie, Podkarpackie, Świętokrzyskie, and Małopolskie Voivodeships) (Figure 1).

The organs used in this study were obtained from animals living in both industrialised and non-industrialised areas (Table 1).

### 2.2. Materials

The study material consisted of liver and kidney samples taken from three game species: roe deer (*Capreolus capreolus*), European red deer (*Cervus elaphus*), and wild boar (*Sus scrofa*). The material was taken from 80 red deer, 75 roe deer, and 80 wild boar. All animals were shot by hunters during a single hunting season (2021) as part of set hunting limits.

The animals were of similar age ranges and body weights within each voivodeship. The mean age of the roe deer was estimated to be 3.5 years, red deer 5.5 years, and wild boar 4 years. The body masses were 13–16 kg for roe deer, 55–80 kg for red deer, and 40–70 kg for wild boar. The age was estimated by the hunters based on the personal and morphological characteristics of the animal (body size, mass and shape, the line of the back, the size and shape of the head and dentition) using a key [22].

The harvested liver and kidney fragments were frozen and stored at −20 °C until analysis.

### 2.3. Analytical Measurements

Homogenised liver and kidney samples (cortical and medullary parts) were mineralised in a mixture of 65% nitric acid (Suprapure, Darmstadt, Germany) and 30% hydrogen peroxide (Suprapure, Merck, Darmstadt, Germany) in an Anton Paar Multiwave microwave oven (Anton Paar Ltd., Hereford, UK). The concentration of Cd in the samples was determined by inductively coupled plasma atomic emission spectrometry (ICP–OES) using a Perkin-Elmer OPTIMA 2000 DV instrument. Measurements were taken along the axial optical path (along the plasma) [23,24,25]. Cadmium concentrations were calculated using calibration curves defined for standards (Merck, Darmstadt. Germany). The detection limit of the instrument was 0.0001 µg/mL (in solution) and 0.005 µg/g (in sample) (Cd).

The analytical procedure was verified based on the Cd levels in the reference material: NCS ZC 71001 (Beef Liver) (China National Analysis Centre for Iron and Steel Beijing China) (Table 2). The samples of reference material (*n* = 15) and reagent samples (blank) (*n* = 15) were also subjected to chemical analysis in parallel with the sample series.

### 2.4. Assessment of Cadmium Intake and Exposure Associated with Liver Consumption

Cadmium intake was assessed according to Warenik-Bany et al. [26] based on exposure by adults (70 kg) and children (23 kg). The subjects were divided into three groups based on the frequency of their consumption of wild game liver: frequent eaters (90 times a year), intermittent eaters (12 times a year), and infrequent eaters (twice a year). A serving of liver was assumed to be 138.4 g for an adult and 111.2 g for a child [27].

Consumer exposure was determined by calculating the estimated daily intake (EDI, mg/kg b.w./day) of Cd based on its concentration in the liver of the tested animals; the analysis was then based on the region, daily average liver consumption, and consumer body weight. In addition, these EDI values were compared with the tolerable weekly intake of Cd: 0.0025 mg/kg b.w. (TWI) [28].

### 2.5. Statistical Analysis

Statistical analysis was performed using Statistica software (StatSoft Inc., ver. 12 StatSoft, Tulsa, OK, USA). The distribution of the variables was tested for normality using the Shapiro–Wilk test and the variation in homogeneity with Laven’s test. Data with a non-normal distribution were then adjusted to a normal distribution by logarithmic transformation and then subjected to a two-way analysis of variance. Significant differences were determined using Duncan’s test on the level of the significance *p* ≤ 0.05.

## 3. Results

### 3.1. Differences in Cd Concentrations in the Analysed Organs with Regard to Species

The highest mean Cd concentrations were noted in wild boar liver and roe deer kidneys, and the lowest concentrations were noted in red deer liver and wild boar kidneys. The mean liver concentrations were 0.159 µg/g w.w. in roe deer, 0.126 µg/g w.w. in red deer, and 0.181 µg/g w.w. in boar, with the respective kidney concentrations being 1.026 µg/g w.w., 0.884 µg/g w.w., and 0.674 µg/g w.w. (Table 3).

**Figure 2:**
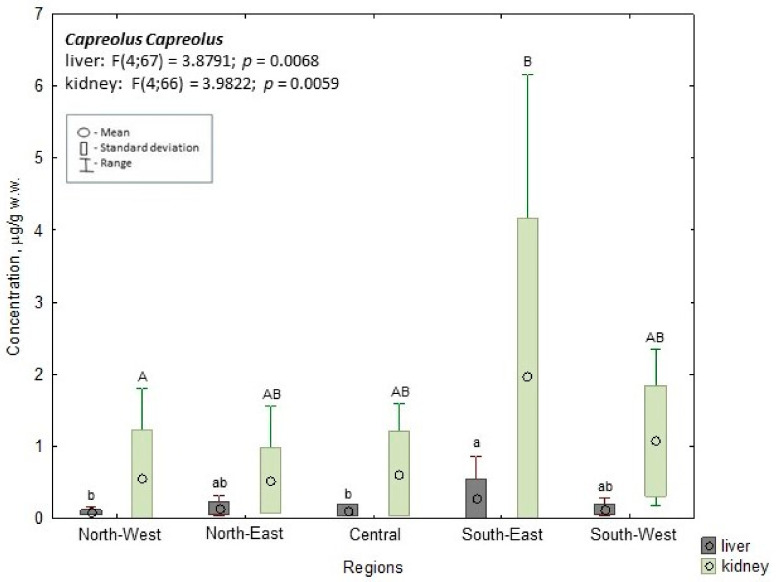
Cd concentration in the liver and kidney of roe deer. a, b—the different characters indicate the significant differences in liver at *p* ≤ 0.05; A, B—the different characters indicate the significant differences in kidney at *p* ≤ 0.05.

In the case of roe deer, the highest mean Cd concentrations (0.275 µg/g w.w.) were found in the southeast region; this value was significantly (*p* ≤ 0.05) higher than the mean values found in roe deer in the northwest (0.082 µg/g w.w.) and central regions (0.114 µg/g w.w., *p* ≤ 0.05). In the kidneys, the highest mean Cd concentration was noted in roe deer from the southeast (1.979 µg/g w.w.); this was significantly higher (*p* ≤ 0.05) than the mean concentrations in the northwest (0.568 µg/g w.w.) (Figure 2).

**Figure 3:**
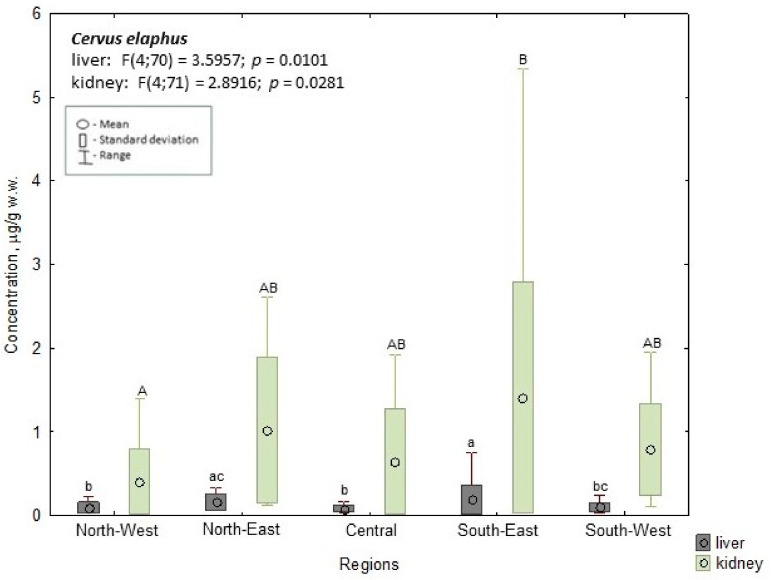
Cd concentration in the liver and kidney of red deer. a, b, c—the different characters indicate the significant differences in liver at *p* ≤ 0.05; A, B—the different characters indicate the significant differences in kidney at *p* ≤ 0.05.

In red deer, the highest mean liver concentration was also found in the southeast (0.192 µg/g w.w.); this value was significantly (*p* ≤ 0.05) higher than the levels found in the central (0.081 µg/g w.w.), northwest (0.094 µg/g w.w.), and southwest regions (0.102 µg/g w.w.; *p* ≤ 0.05). In the northeast region, the mean Cd concentration (0.161 µg/g w.w.) was significantly higher than in the central (*p* ≤ 0.05) and northwest regions (*p* ≤ 0.05). The highest mean concentrations in the kidneys were found in the southeast region (1.411 µg/g w.w.); this value was significantly higher (*p* ≤ 0.05) than the mean concentrations in the northwest region (0.407 µg/g w.w.) (Figure 3).

**Figure 4:**
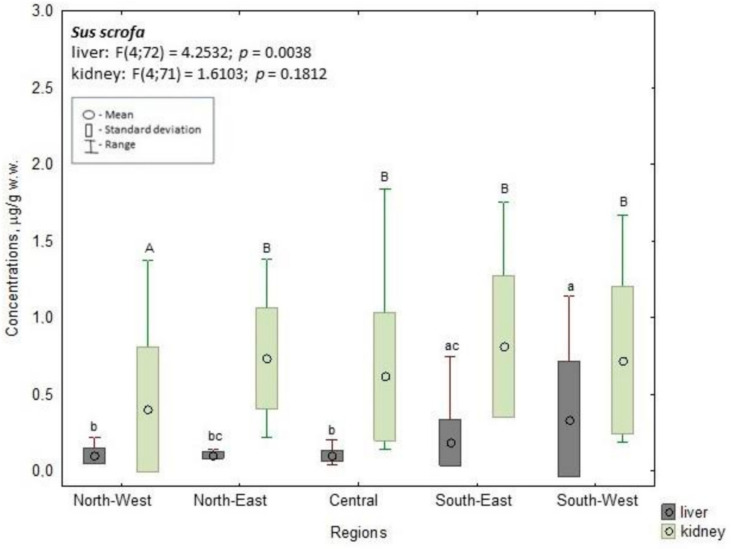
Cd concentration in the liver and kidney of wild boar. a, b, c—the different characters indicate the significant differences in liver at *p* ≤ 0.05; A, B—the different characters indicate the significant differences in kidney at *p* ≤ 0.05.

Among wild boar, the highest liver concentrations were observed in the southwest region (0.337 µg/g w.w.); this value was three times higher than those noted in the central (0.098 µg/g w.w.), northwest (0.099 µg/g w.w.), and northeast regions (0.104 µg/g w.w.). These differences were statistically significant (*p* ≤ 0.05). In contrast, the concentrations in the southeast region (0.186 µg/g w.w.) were significantly higher than those in the central and northwest regions (*p* ≤ 0.05). In wild boar kidneys, significantly higher (*p* ≤ 0.05) mean Cd concentrations were found in animals from the southeast region (0.811 µg/g w.w.) than in the northwest region (0.402 µg/g w.w.), i.e., twice as high. The mean concentrations in the northwest region were also significantly lower (*p* ≤ 0.05) than those found in the southwest (0.722 µg/g w.w.), northeast (0.732 µg/g w.w.), and central regions (0.616 µg/g w.w., *p* ≤ 0.05) (Figure 4).

### 3.2. Differences in Cd Concentrations in the Tested Animal Species with Regard to Their Region

The Cd concentrations were found to vary according to region. In the liver, the highest mean concentrations were found in the southeast for roe deer and red deer and in the southwest for wild boar. In the kidneys, the highest mean concentrations were found in the southeast for all species (Table 3).

Among the regions, the highest mean Cd concentration was found in the livers from the southeast (0.217 µg/g w.w.), and the lowest concentration was found in the northwest (0.091 µg/g w.w.). The highest mean concentration was recorded in the liver of wild boar from the southwest (0.337 µg/g w.w.); this value was significantly higher than those found in the livers of red deer (0.102 µg/g w.w.; *p* ≤ 0.05) and roe deer (0.131 µg/g w.w.; *p* ≤ 0.05) from this region (Table 4).

As with the liver, the highest mean kidney Cd concentrations were also found in the southeast (1.400 µg/g w.w.), and the lowest were found in the northwest region (0.459 µg/g w.w.). In the southeast, the highest Cd levels were noted in roe deer (1.979 µg/g w.w.), these levels being more than double those of wild boar (0.811 µg/g w.w.) from this region (Table 4).

### 3.3. Comparison of Estimated Daily Intake (EDI) of Cd with Weekly Tolerable Intake (TWI)

The estimated daily intake (EDI) of Cd for adults and children resulting from liver consumption and the percentage of Cd in the total weekly intake (TWI) are presented in Table 5.

The highest intake of Cd was 0.000402 mg/kg b.w., while the lowest was 0.000001 mg/kg b.w., representing 112.49% and 0.25% of the TWI dose, respectively.

The highest EDI value was associated with the consumption of wild boar liver by both adults (0.000088 mg/kg b.w.) and children (0.000216 mg/kg b.w.), representing 24.71 and 60.42% of the TWI dose, respectively. In contrast, the lowest EDI was observed for deer liver consumption: adult (0.000061 mg/kg b.w.); children (0.000150 mg/kg b.w.), representing 17.20 and 42.06% of TWI, respectively.

The highest EDI was associated with the consumption of roe deer and red deer liver from the southeast region and wild boar from the southwest region. The lowest EDIs were associated with red deer liver from the northwest and red deer and wild boar liver from the central region.

It was observed that the EDI values of Cd consumed in game liver were 2 to 2.5 times higher in children than in adults. However, among children, the TWI dose was only exceeded for the frequent consumption (90 times a year) of wild boar liver from the southwest region (0.000402 mg/kg b.w.), which was 112% of the TWI. For adults, TWI values were not exceeded in any analysed region (Table 5).

## 4. Discussion

### 4.1. Cadmium Concentrations in the Livers and Kidneys of Examined Animals

The condition of soils, sediments, and surface waters is influenced by natural processes and human activities. In terms of geochemistry, Poland can be divided into a northern region (lowlands) and a southern region (uplands), which differ in their geochemical background. In the case of Cd, a greater geochemical background level is found in Southern Poland, which is related to the fact that soils have been formed on magmatic and metamorphic rocks. Indeed, while the Cd concentrations of most soils in the country do not exceed 0.5 mg/kg, this level can be greater than 1 mg/kg in the south [29].

Our findings also confirm that the southern part of Poland bears the highest Cd burden of all the regions. This can be caused not only by natural processes, but also by anthropogenic factors, such as the metallurgical, smelting, and mining industries, which are strong sources of Cd emissions and concentrated in the southern regions of Poland. Biernacka and Małuszyński [30] report significantly higher Cd levels in soils in areas with a high rate of industrial pollution in Southern Poland (1.40–3.20 mg/kg d.w.) compared with northeastern areas (0.25–0.64 mg/kg d.w.) considered to be unpolluted. Similar results were obtained by Dudka [31], who found higher Cd concentrations in the soils of Southwestern Poland (0.52 mg/kg) than in the northeastern regions (0.27 mg/kg).

The cadmium concentration in the livers of the studied animals is a very good indicator of current Cd intake. Our findings indicate that animals from the southern regions of Poland (SE and SW) are the most exposed to Cd, which would correspond to the high Cd concentrations in the soil in this area. Conversely, it was expected that the animals from the northeastern region, an industry-free area considered the cleanest region in Poland, would demonstrate the lowest Cd concentrations. However, the results obtained for roe and red deer were comparable to those obtained in industrialised areas. This could be due to the point source contamination of foraging areas, or elevated geochemical background levels at this location. It should also be noted that Cd residues may be present in the bark of trees, following the earlier use of cadmium chloride to protect the forest stand. Indeed, lower Cd levels were noted in the livers of wild boar, which are more trophically related to the soil environment, compared to roe deer and deer, which eat tree bark. The Cd concentrations in the liver in each region followed the following order according to test animal: in roe deer: southeast > northeast > southwest > central > northwest; in red deer: southeast > northeast > southwest > northwest > central; in wild boar: southwest > southeast > northeast > northwest > central.

The cadmium concentration in the kidney reflects the long-term uptake of Cd by different pathways. Our results indicate the greatest long-term exposure in the southeast region; in addition, high levels are found in roe deer from the southwest, and more surprisingly, in red deer and wild boar from the northeast. As with the liver results, the high concentrations observed in the northeast region are unexpected, and these may be related to the specific physico-chemical characteristics of this region than any human influence.

In all regions of Poland, higher amounts of cadmium were recorded in the kidneys than the livers, which was also confirmed elsewhere [32,33,34]. It can be assumed that this situation is related to long-term exposure to low concentrations of Cd in food. As reported by Jin et al. [35], acute high exposure results in Cd accumulation mainly in the liver; in contrast, long-term exposure to low dietary concentrations results in accumulation in the kidneys, especially in the cortical part. Pérez-López et al. [36] propose that Cd concentrations greater than 1 mg/kg d.w. in the liver signal an increase in environmental exposure.

In the present study, the Cd concentrations in the liver and kidneys of roe deer from the southeastern region were comparable with those obtained by Lech and Gubala [37] in the area of Krakow, also in the southeast; the Cd concentration was 0.27 mg/kg w.w. in the liver and 1.7 mg/kg w.w. in the kidneys. High concentrations were noted in roe deer from Southeast Poland by Krupa and Szmulik [38]. Compared to the present findings in the same area, the authors report twice as much Cd in the liver (0.49 mg/kg w.w.) and five times as much in the kidney (10.0 mg/kg w.w.).

Wild boar from the southeastern provinces exhibited lower liver and kidney Cd concentrations than those obtained by Rudy [39] in 2002–2006 in the Podkarpackie Province (liver 0.290 to 0.417 mg/kg w.w.).

In the southwest region, in roe deer, Cd concentrations were 0.131 µg/g w.w. in the liver and 1.075 µg/g w.w. in the kidney. A previous study of concentrations in game animals in this region by Kucharczak et al. [40] found almost twice the present Cd level in the liver of roe deer (0.24 mg/kg w.w.), but half the present level was found in the kidney (0.46 mg/kg w.w.). Similar results were obtained by Kucharczak et al. [41] in roe deer from areas adjacent to the Turów Power Plant and Mine (Bogatynia), where the concentrations were 0.279 mg/kg w.w. in the liver and 0.589 mg/kg w.w. in the kidneys.

In the southwest region, the tested wild boar demonstrated three times the liver Cd concentration compared to the other tested animal species from this region, i.e., in an area with a high concentration of metallurgical, smelting, and mining industries. This situation is related to the nutritional differences found between the tested animal species. Reeves and Chaney [42] report that a diet high in protein and low in fibre increases the absorption capacity of Cd. Mosses are also characterised by a very intensive uptake of Cd (8–340 mg/kg), and wild boar take up larger amounts of moss when rutting [43]. According to Jasiewicz and Antonkiewicz [44], Cd accumulates in greater quantities in plant roots than stems. In addition, the long-term and widespread use of artificial fertilisers (e.g., superphosphates) represents a significant source of cadmium in the environment, resulting in ongoing contamination. In addition to forest and meadow vegetation, wild boar very often use field crops; these make up a third of the diet of a wild boar, with cereals and potatoes occurring in equal amounts.

Similar findings regarding Cd levels in wild boar liver were obtained by Kucharczak et al. [40] in the vicinity of Wrocław (0.32 mg/kg w.w.). However, the Cd concentration in the kidneys was three times higher than in the present study (2.22 mg/kg w.w.).

In the central region, all three species demonstrated lower Cd levels in both the liver and kidneys compared to previous studies. This may be associated with the significant reduction in the use of Cd in industry. Our findings are seven times lower than those recorded in roe deer liver (0.79 µg/g w.w.) and three times higher (0.33 µg/g w.w.) than those previously noted in wild boar liver in this part of Poland [45].

The Cd levels in the liver and kidneys of roe deer from the northeastern provinces vary considerably between studies. A previous study of roe deer from Northeast Poland obtained three-times higher liver Cd concentrations (0.37 mg/kg w.w.) and four-times higher kidney concentrations (2.29 mg/kg w.w.) compared to our present findings (Zasadowski and Wyszyńska) [46]. Falandysz et al. [32] report slightly higher Cd concentrations in the liver of red deer (0.19 mg/kg w.w.) in the northeast (Warmia and Mazury region) than in our study; however, they also note the levels in the kidneys to be twice as high (2.2 mg/kg w.w.). Kośla et al. [47] found bison living in the Białowieża Forest in the northeast to have approximately six times higher liver Cd levels (0.83 mg/kg w.w.) compared to roe deer, red deer, and wild boar from the northeast. However, the Cd concentrations in the kidneys (1.82 mg/kg w.w.) were three times higher than those recorded in roe deer, almost twice those of red deer, and around 2.5 times higher than wild boar in the present study.

The Cd concentrations of the organs from game animals also vary between other European countries. Significant amounts have been recorded in the south of Europe, especially Croatia and Slovenia. Compared to our present findings, higher concentrations were found in the liver (0.568 mg/kg w.w.) and kidney (4.905 mg/kg w.w.) regarding roe deer in an area in Northwestern Croatia [48]. Pokorny and Ribarič-Lasnik [49] also found significantly higher Cd levels in roe deer from the northern part of Slovenia; the Cd concentrations ranged from 1.06 to 3.93 mg/kg w.w. in the liver, depending on age, and from 2.91 to 22.73 mg/kg w.w. in the kidneys.

The Cd levels of the red deer kidneys in the present study are comparable with those obtained in eastern Croatia (0.944 mg/kg w.w.) by Lazarus et al. [50]. In addition, a study of red deer from seven different regions of Croatia identified Cd levels ranging from 1.04 to 2.54 mg/kg w.w. in the kidney, and from 0.11 to 0.20 mg/kg w.w. in the liver [51]. Elsewhere, a Slovakian study recorded twice the Cd levels (0.258 mg/kg w.w.) in the liver and three times the levels (2.387 mg/kg w.w.) in the kidney compared to the present study [52,53].

Our present findings were similar to those found in the liver (0.16 mg/kg w.w.) and kidneys (0.68 mg/kg w.w.) of wild boar in the northwestern part of Russia [54]. In contrast, the Cd concentrations in wild boar kidneys in the eastern areas of Croatia were four times higher than in the present study. The authors [55] report that the kidney Cd concentrations ranged from 2.65 to 2.91 mg/kg w.w. depending on the region of the country. In contrast, Bilandžic et al. [56] found the levels to range from 0.866 to 4.58 mg/kg w.w. in wild boar from the northeastern regions of Croatia. An earlier study by Bilandžic et al. [51] in four regions of Croatia found Cd concentrations to range from 3.47 to 5.98 mg/kg w.w. in the kidneys, and from 0.30 to 0.49 mg/kg w.w. in the liver. Gašparík et al. [57] report higher Cd concentrations in the organs of wild boar in Slovakia, i.e., 0.474 µg/g w.w. in the liver and 2.73 µg/g w.w. in the kidneys compared to the present study.

In Italy, Danieli et al. [58] obtained Cd concentrations of 0.084 mg/kg w.w. in wild boar liver, i.e., half the value found in the present study. Similar results in wild boar liver (0.085 mg/kg w.w.) were reported in Central Italy by Amici et al. [11]. However, the authors report almost twice the Cd concentration in wild boar kidney (1.052 mg/kg w.w.) compared to the present study.

**Figure 5:**
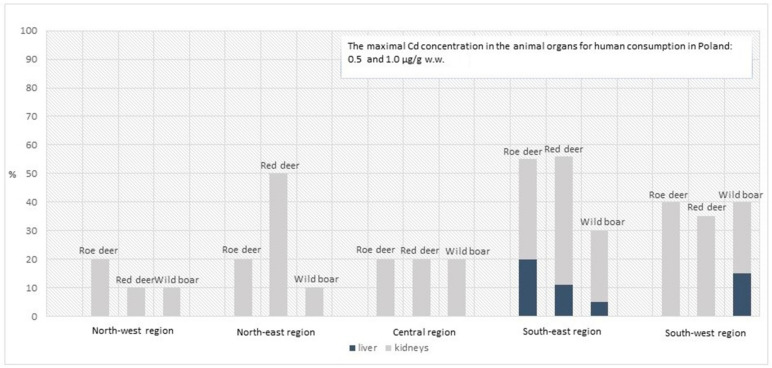
The number of animals and the percentage of samples with the exceeded maximal Cd concentration in livers and kidneys (0.5 and 1.0 µg/g w.w. [59]).

In Poland, the maximum permissible concentrations of Cd in the liver and kidney were 0.5 and 1.0 µg/g w.w., respectively [59]. A negligible number of such samples were found in the livers of all the tested species in the southeastern region, and one in the liver of wild boar from the southwestern region. In contrast, all animals throughout the country demonstrated excessive Cd levels in the kidney, with the highest proportions being observed in the southwest and southeast regions, as well as in red deer from the northeast region. The numbers of animals with excessive Cd concentrations, and their percentage occurrence, are given in Figure 5.

### 4.2. EDI and TWI of Cd in Association with Liver Consumption

Any heavy metals released in excessive quantities into the environment are also incorporated into the food chain. As the main source of Cd for the human body is food, there is a clear need to study the levels of toxic elements in food and to monitor them. Within the European Union, such food safety surveillance is carried out by the European Food Safety Agency (EFSA), which has set the tolerable weekly intake (TWI) for Cd at 2.5 µg/kg b.w. [28]. Our data indicate that TWI was not exceeded in the case of adults, while in children, TWI was exceeded only in the case of the frequent consumption of wild boar liver from the southwest region.

Our findings indicate that the daily Cd intake (EDI) of children with game animal liver was 2–2.5 times higher than that of adults. Similar results were reported by Pilarczyk et al. [27] for roe deer and wild boar in industrialised areas of Ukraine.

Regarding wild boar liver, the EDI values for adults and children in the northern and central regions were comparable to those obtained by Pilarczyk et al. [27]; however, those obtained in the southern regions were twice as high, and those for children from the southwest region were four times higher than Pilarczyk et al. [27]. In Slovakia, Gašparík et al. [57] note that the provisional tolerated weekly intake (PTWI) of Cd was temporarily exceeded in the liver and kidneys of wild boar; however, the muscle tissue appeared to be safe for consumption by consumers. Pilarczyk et al. [27] report risk quotients (HQs) and hazard indexes (HIs) of less than one for wild game livers, indicating a low probability of risk to consumer health. In contrast, Kicinska et al. [60] obtained a higher HI (>1) for the liver of game animals than for livestock.

## 5. Conclusions

Clear differences in the Cd concentrations of the organs of free-living animals were found with regard to their region of origin. Regions in Southern Poland proved to be the most burdened with Cd. The reason for this may be the intensive industrial activities carried out in these areas. Our study also showed high Cd levels in the northeast, an area where no industrial plants are located; this demonstrates that the absence of industrial plants is not a decisive factor in assessing the contamination of a region, and that food from animals in such areas cannot automatically be classified as potentially healthy for consumers.

Excessive Cd levels were found in the kidneys of test animals throughout the country. The highest proportion of such samples was found in the kidneys of animals from the southwest and southeast regions, and deer from the northeast region.

A small number of liver samples with excessive Cd concentrations were recorded among animals in the southeast region, and wild boar from the southwest. TWI levels were only found to be exceeded in children who frequently consumed wild boar liver from the southwest.

## Figures and Tables

**Table 1:** Characteristics of the test area.

Region	Voivodeships	Industry
Northeast	Podlaskie, Warmińsko-mazurskie	None; area known as the green lungs of Poland
Northwest	Pomorskie, Zachodniopomorskie	Chemical plants producing phosphate fertilisers; refineries producing fuels, oils, and lubricants; thermal power plants; shipbuilding industry
Southwest	Dolnośląskie, Opolskie, Śląskie, Lubuskie	mines, steel mills, copper ore deposits; energy, machinery, chemicals, fuel and energy, chemical and automotive industries
Central	Kujawsko-pomorskie, Łódzkie, Mazowieckie, Wielkopolskie	Chemical plants, production of lime for fertiliser, combined heat and power plants, paint and varnish production, nitrogen plants; mining, coal power, machinery and electrical engineering, metallurgy, printing, electronics, automotive industry, transportation
Southeast	Świętokrzyskie, Małopolskie, Lubelskie, Podkarpackie	Mining, oil refining, metallurgy, ceramics, foundry, energy production

**Table 2:** The concentration of Cd in the NCS ZC 71001 reference material (bovine liver, µg/g dry matter, mean ± standard deviation).

Element	Reference Material NCS ZC 71001		Recovery (%)
	Certified Concentration (µg/g Dry Matter)	Obtained Concentration (µg/g Dry Matter)	
Cd	0.388	0.387 ± 0.02	99.7%

**Table 3:** The mean concentration of Cd in the livers and kidneys of studied animals in accordance to geographic location.

Region	Cd Concentration (µg/g w.w.)											
	*Capreolus capreolus*				*Cervus elaphus*				*Sus scrofa*			
	*n*	$\bar{X}$	SD	Range	*n*	$\bar{X}$	SD	Range	*n*	$\bar{X}$	SD	Range
Liver												
Northwest	10	0.082 ^b^	0.035	0.048–0.156	10	0.094 ^b^	0.055	0.046–0.220	10	0.099 ^b^	0.052	0.056–0.218
Northeast	10	0.141 ^ab^	0.092	0.034–0.317	10	0.161 ^ac^	0.099	0.060–0.333	10	0.104 ^bc^	0.022	0.082–0.140
Central	20	0.114 ^b^	0.074	0.052–0.388	20	0.081 ^b^	0.040	0.039–0.171	20	0.098 ^b^	0.038	0.045–0.205
Southeast	20	0.275 ^a^	0.282	0.057–0.863	20	0.192 ^a^	0.183	0.068–0.752	20	0.186 ^ac^	0.150	0.073–0.742
Southwest	15	0.131 ^ab^	0.077	0.043–0.276	20	0.102 ^bc^	0.047	0.036–0.237	20	0.337 ^a^	0.385	0.066–1.140
Total	75	0.159	0.173	0.034–0.863	80	0.126	0.107	0.036–0.752	80	0.181	0.223	0.045–1.140
Kidneys												
Northwest	10	0.568 ^A^	0.661	0.112–1.801	10	0.407 ^A^	0.403	0.130–1.391	10	0.402 ^A^	0.408	0.130–1.371
Northeast	10	0.524 ^AB^	0.452	0.136–1.558	10	1.024 ^AB^	0.867	0.121–2.613	10	0.732 ^B^	0.331	0.221–1.379
Central	20	0.617 ^AB^	0.585	0.042–2.376	20	0.647 ^AB^	0.657	0.108–1.922	20	0.616 ^B^	0.420	0.139–1.839
Southeast	20	1.979 ^B^	2.308	0.173–6.162	20	1.411 ^B^	1.458	0.096–5.340	20	0.811 ^B^	0.487	0.352–1.754
Southwest	15	1.075 ^AB^	0.821	0.169–2.354	20	0.790 ^AB^	0.553	0.107–1.952	20	0.722 ^B^	0.510	0.191–1.670
Total	75	1.026	1.376	0.042–6.162	80	0.884	0.930	0.096–5.340	80	0.674	0.456	0.130–1.839

*n*—number of tested animals; $\bar{X}$—mean; SD—standard deviation; w.w.—wet weight; ^a,b,c^—the different characters indicate the significant differences in the liver in particular species at *p* ≤ 0.05; ^A,B^—the different characters indicate the significant differences in kidney in particular species at *p* ≤ 0.05.

**Table 4:** The mean concentration of Cd in the livers and kidneys of examined animals in accordance to geographic region.

Animal	Region	*n*	Cd Concentration (µg/g w.w.)					
			Liver			Kidneys		
			$\bar{X}$	SD	Range	$\bar{X}$	SD	Range
*Capreolus capreolus*	Northwest	10	0.082 ^a^	0.035	0.048–0.156	0.568 ^A^	0.661	0.112–1.801
*Cervus elaphus*		10	0.094 ^a^	0.055	0.046–0.220	0.407 ^A^	0.403	0.130–1.391
*Sus scrofa*		10	0.099 ^a^	0.052	0.056–0.218	0.402 ^A^	0.408	0.130–1.371
Total		30	0.091	0.049	0.046–0.220	0.459	0.430	0.112–1.801
*Capreolus capreolus*	Northeast	10	0.141 ^a^	0.092	0.034–0.317	0.524 ^A^	0.452	0.136–1.558
*Cervus elaphus*		10	0.161 ^a^	0.099	0.060–0.333	1.024 ^A^	0.867	0.121–2.613
*Sus scrofa*		10	0.104 ^a^	0.022	0.082–0.140	0.732 ^A^	0.331	0.221–1.379
Total		30	0.135	0.088	0.034–0.333	0.752	0.630	0.121–2.613
*Capreolus capreolus*	Central	20	0.114 ^a^	0.074	0.052–0.388	0.617 ^A^	0.585	0.042–2.376
*Cervus elaphus*		20	0.081 ^a^	0.040	0.039–0.171	0.647 ^A^	0.657	0.108–1.922
*Sus scrofa*		20	0.098 ^a^	0.030	0.045–0.205	0.616 ^A^	0.420	0.139–1.839
Total		60	0.098	0.054	0.039–0.388	0.626	0.550	0.042–2.376
*Capreolus capreolus*	Southeast	20	0.275 ^a^	0.282	0.057–0.863	1.979 ^A^	2.308	0.173–6.162
*Cervus elaphus*		20	0.192 ^a^	0.183	0.068–0.752	1.411 ^A^	1.458	0.096–5.340
*Sus scrofa*		20	0.186 ^a^	0.150	0.073–0.742	0.811 ^A^	0.487	0.352–1.754
Total		60	0.217	0.209	0.057–0.863	1.400	1.650	0.096–6.162
*Capreolus capreolus*	Southwest	15	0.131 ^a^	0.077	0.043–0.276	1.075 ^A^	0.821	0.169–2.354
*Cervus elaphus*		20	0.102 ^a^	0.047	0.036–0.237	0.790 ^A^	0.553	0.107–1.952
*Sus scrofa*		20	0.337 ^b^	0.385	0.066–1.140	0.722 ^A^	0.510	0.191–1.670
Total		55	0.195	0.260	0.036–1.140	0.839	0.623	0.107–2.354

*n*—number of tested animals; $\bar{X}$—mean; SD—standard deviation; w.w.—wet weight; ^a,b^—the different characters indicate the significant differences in the liver in a particular region, ^A^—in the kidney at *p* ≤ 0.05.

**Table 5:** Estimated daily intake and tolerable weekly intake of Cd in association with liver consumption.

Region	EDI—Estimated Daily Intake (mg/kg b.w.)						% TWI					
	Frequent Consumption (90 Times/Year)		Periodic Consumption (12 Times/Year)		Occasional Consumption (2 Times/Year)		Frequent Consumption (90 Times/Year)		Periodic Consumption (12 Times/Year)		Occasional Consumption (2 Times/Year)	
	Adult	Children	Adult	Children	Adult	Children	Adult	Children	Adult	Children	Adult	Children
*Capreolus capreolus*												
Northwest	0.000040	0.000098	0.000005	0.000013	0.000001	0.000002	11.19	27.37	1.49	3.65	0.25	0.61
Northeast	0.000069	0.000168	0.000009	0.000022	0.000002	0.000004	19.25	47.07	2.57	6.28	0.43	1.05
Central	0.000056	0.000136	0.000007	0.000018	0.000001	0.000003	15.56	38.05	2.07	5.07	0.35	0.85
Southeast	0.000134	0.000328	0.000018	0.000044	0.000003	0.000007	37.54	91.79	5.01	12.24	0.83	2.04
Southwest	0.000064	0.000156	0.000009	0.000021	0.000001	0.000003	17.88	43.73	2.38	5.83	0.40	0.97
Total	0.000078	0.000190	0.000010	0.000025	0.000002	0.000004	21.70	53.07	2.89	7.08	0.48	1.18
*Cervus elaphus*												
Northwest	0.000046	0.000112	0.000006	0.000015	0.000001	0.000002	12.83	31.38	1.71	4.18	0.29	0.70
Northeast	0.000078	0.000192	0.000010	0.000026	0.000002	0.000004	21.98	53.74	2.93	7.17	0.49	1.19
Central	0.000039	0.000097	0.000005	0.000013	0.000001	0.000002	11.06	27.04	1.47	3.61	0.25	0.60
Southeast	0.000094	0.000229	0.000012	0.000031	0.000002	0.000005	26.21	64.09	3.49	8.55	0.58	1.42
Southwest	0.000050	0.000122	0.000007	0.000016	0.000001	0.000003	13.92	34.05	1.86	4.54	0.31	0.76
Total	0.000061	0.000150	0.000008	0.000020	0.000001	0.000003	17.20	42.06	2.29	5.61	0.38	0.93
*Sus scrofa*												
Northwest	0.000048	0.000118	0.000006	0.000016	0.000001	0.000003	13.51	33.05	1.80	4.41	0.30	0.73
Northeast	0.000051	0.000124	0.000007	0.000017	0.000001	0.000003	14.20	34.72	1.89	4.63	0.32	0.77
Central	0.000048	0.000117	0.000006	0.000016	0.000001	0.000003	13.38	32.71	1.78	4.36	0.30	0.73
Southeast	0.000091	0.000222	0.000012	0.000030	0.000002	0.000005	25.39	62.09	3.39	8.28	0.56	1.38
Southwest	0.000164	0.000402	0.000022	0.000054	0.000004	0.000009	46.00	112.49	6.13	15.00	1.02	2.50
Total	0.000088	0.000216	0.000012	0.000029	0.000002	0.000005	24.71	60.42	3.29	8.06	0.55	1.34

## Data Availability

The data presented in this study are available on request from the corresponding author.
